# Effect of stem cell transplantation for B-cell malignancies on disease course of associated polyneuropathy

**DOI:** 10.1007/s00415-012-6463-0

**Published:** 2012-03-08

**Authors:** A. C. J. Stork, W. L. van der Pol, D. van Kessel, H. M. Lokhorst, N. C. Notermans

**Affiliations:** 1 Department of Neurology, Rudolf Magnus Institute of Neuroscience, University Medical Center, Utrecht, The Netherlands; 2 Department of Hematology, University Medical Center, Utrecht, The Netherlands; 3 Department of Neuromuscular Diseases, Rudolf Magnus Institute of Neuroscience, P.O. Box 85500, 3508 GA Utrecht, The Netherlands

**Keywords:** SCT, Polyneuropathy, B-cell malignancy

## Abstract

B cell dyscrasias are often refractory to medical treatments, and hematological stem cell therapy (SCT) may be warranted. It is not clear whether an associated polyneuropathy may also profit from SCT. In exceptional cases SCT has been tried in patients with monoclonal gammopathy and progressive polyneuropathy refractory to medical treatments. In a cohort of 225 patients with monoclonal gammopathy and polyneuropathy, we selected the six patients who underwent SCT and retrospectively examined the effects of SCT on the disease course of the associated polyneuropathy. In all patients except one, the indication for SCT was hemato-oncological (multiple myeloma in 4 patients and primary AL amyloidosis in 1). The remaining patient had an IgG monoclonal gammopathy of undetermined significance and a progressive and painful polyneuropathy for which she was treated with SCT. SCT led to improvement of motor scores and autonomic symptoms in one patient; three patients experienced improvement of neuropathic pain or sensory deficits but showed further progression of weakness. One patient showed no improvement at all. One patient died within 100 days after SCT. In conclusion, SCT as a treatment of refractory hematological malignancy may occasionally have a positive effect on the associated progressive polyneuropathy, although the benefits are very limited and the treatment-related mortality is high.

## Introduction

B cell dyscrasias are associated with polyneuropathies. A variety of pathogenetic mechanisms, including cytokine-mediated damage, nerve infiltration, synthesis and deposition of noxious monoclonal proteins or binding of monoclonal antibodies to nerve constituents, may cause neuropathy in patients with B cell disorders. The disease course of these polyneuropathies may be mild, but can also be very debilitating, causing severe sensorimotor deficits and autonomic dysfunction [1, 2].

Unfortunately, polyneuropathy associated with monoclonal gammopathy is often refractory to treatment. Intravenous immunoglobulins (IVIg) and the anti-CD20 monoclonal antibody rituximab may ameliorate the disease course in a minority of patients [3, 4]. Hematological stem cell therapy (SCT) is a well-established therapy used for refractory hematological malignancies. SCT has been reported to attenuate the progressive disease course of patients with polyneuropathy with organomegaly, endocrinopathy, M-protein and skin changes (POEMS) [5, 6], chronic inflammatory demyelinating polyneuropathy (CIDP) [7, 8], primary AL amyloidosis [9], and recently in a small series of patients with IgG MGUS- or MM-associated neuropathy [10], but it is unknown whether SCT may represent a rescue therapy for a wider range of patients with polyneuropathy and B cell dyscrasias and whether the possible beneficial effects outweigh risks associated with SCT.

Here we describe six patients with malignancy-associated polyneuropathy receiving SCT primarily for the treatment of their hematological disorders and report on the effects on the neuropathy.

## Patients and methods

### Patient characteristics

Six patients (2.7%) from a cohort of 225 patients [11] with polyneuropathy and B cell dyscrasia underwent SCT. The primary indication for SCT was the hematological malignancy in all patients with the exception of patient 5, in whom improvement of the polyneuropathy was the main goal. Clinical data and data from nerve conduction studies were collected prospectively following a standardized protocol that has been in use for this patient group since 1985. These data were analyzed retrospectively to asses the effect of SCT on the course of the polyneuropathy. Patient characteristics are summarized in Table 1. All patients presented with complaints consistent with polyneuropathy, with B cell dyscrasia found during the neuropathy work-up. Four patients had multiple myeloma (MM) unresponsive to other treatments (patients 1, 2, 3 and 6), one had light chain (AL) amyloidosis, and one patient had an IgG MGUS and a rapidly progressive demyelinating polyneuropathy despite treatment with IVIg and glucocorticosteroids (patient 5). With the exception of patient 1, who had a monozygous twin brother who acted as a donor, all patients were treated with autologous SCT. All transplantations were carried out in the Utrecht Medical Center, a tertiary referral center for hemato-oncology and (autologous) stem cell transplantation. Institutional morbidity and mortality lie well within international standards, as published elsewhere (10% treatment related mortality in MM patients) [11].

**Table 1:** Patients’ age at first visit in our neuromuscular clinic, paraprotein, hematological diagnosis and predominant clinical manifestation of the polyneuropathy

Patient	Age	Paraprotein	Hematological diagnosis	Polyneuropathy	Motor sum score		Sensory sum score		Rankin score		Clinical effect of SCT on polyneuropathy
					Before	After	Before	After	Before	After	
1	44	IgAλ	Multiple myeloma	Motor	122	137	54	56	2	2	Improvement
2	60	IgAλ	Multiple myeloma	Sensorimotor	132	128	44	40	2	2	Stabilization
3	59	IgGκ	Multiple myeloma	Sensorimotor	108	(*)	24	(*)	3	(*)	Rapid further progression, death
4	43	IgGλ	AL amyloidosis	Sensory and autonomic	140	137	46	44	3	2	Improvement in autonomic neuropathy
5	58	IgGλ	MGUS	Sensorimotor	112	110	27	31	4	4	Stabilization
6	48	IgDλ	Multiple myeloma	Sensorimotor	138	136	50	50	2	2	Stabilization

Motor sum score, sensory sum score, modified ranking score, before and after treatment. Overall clinical result. (*) died before 1 year follow-up

Neurological examinations before and 1 year after stem cell transplantation were performed using a standardized protocol, described elsewhere [12]. Motor function was expressed using the Modified Medical Research Council (MRC) motor sum score of 14 muscle groups in both arms and legs, with a maximum score of 140 points. Sensory function was expressed as a sensory composite sum score consisting of scores for sense of touch (0–4), pain (0–4) and vibration (0–4) in both arms and legs, and position sense (0–2) in both legs with a maximum of 56 points [12]. The modified Rankin score, (ranging from 0 for no symptoms at all to 5 for severely disabled and bedridden and 6 for dead) was used to quantify functional disability.

All patients underwent systematic nerve conduction studies using a previously described protocol [12]. Demyelination was defined according to previously established criteria. Nerve conduction studies that showed a reduction of CMAP amplitudes to less than 20% of normal values in at least two nerves but did not meet the criteria for demyelination were scored as axonal [13].

Other causes of polyneuropathy than monoclonal gammopathy were excluded following a standardized protocol [12].

## Case histories and results (Table 1)

Patient 1 was diagnosed with MM with IgA monoclonal gammopathy and had a progressive, predominantly motor neuropathy, with severe weakness, hypesthesia and paresthesia in the lower legs and hypesthesia in the fingertips. Nerve conduction studies showed distal demyelinisation in arms and legs. He was first treated with melfalan and dexamethasone and then, after pre-treatment with cyclophosphamide and total body irradiation, underwent allogenic stem cell transplantation with stem cells from his HLA-identical twin brother 18 months after the first manifestation of polyneuropathy. SCT was complicated by mild graft versus host disease. One year after transplantation, strength in his legs had virtually normalized (MRC motor sum score improved from 122 to 137 out of 140).

Neurological deficits further improved slightly in the second year after SCT and then remained unchanged until 11 years after transplantation, when an exacerbation of MM was preceded by progression of sensory complaints and weakness. Nerve conduction studies abnormalities met the criteria for axonal polyneuropathy. After a second SCT with the original stem cell harvest, weakness and sensory deficits did not deteriorate any further.

Patient 2 was diagnosed with MM, an IgA-lambda gammopathy, and had a sensorimotor axonal polyneuropathy, with muscle atrophy, weakness, hypesthesia and paresthesia in both lower legs and mild weakness of the intrinsic hand muscles. MM was first treated with two cycles of intermediate dose melfalan. Autologous stem cell transplantation was then performed after pre-treatment with cyclophosphamide and total body irradiation. Mucositis was the most important complication of SCT. SCT was performed 1 year after diagnosis of the polyneuropathy and 3 years after the first neuropathic complaints. After transplantation MRC motor sum scores declined a further four points to 128/140, while the sensory sum score declined from 44 to 40 out of 56. Follow-up during 5 years showed no further changes in neurological examination, and nerve conduction studies did not show further progression.

Patient 3 had MM, an IgG-kappa gammopathy and a rapidly progressive sensorimotor polyneuropathy. Nerve conduction studies met the criteria for axonal polyneuropathy. Marked weakness was present distally in arms and legs, together with sock-and-glove patterned hypesthesia and sensory ataxia. Ambulation was lost during the diagnostic work-up. After three cycles of adriamycin/dexamethasone and two cycles of intermediate dose melfalan, autologous stem cell transplantation 30 months after the first manifestation of polyneuropathy seemed to stabilize the disease course for the duration of 1 month, but 2 months after transplantation the polyneuropathy deteriorated and caused severe weakness of respiratory and bulbar muscles. Three months after transplantation the patient died of respiratory insufficiency.

Patient 4 had primary amyloidosis with light chain gammopathy and progressive neuropathy with sensory ataxia and autonomic dysfunction leading to impotence, orthostatic hypotension, and loss of bowel and bladder control. Nerve conduction studies met the criteria for axonal polyneuropathy. He was also treated with adriamycin, dexamethasone and melfalan, followed by autologous stem cell transplantation. After SCT this patient developed herpes labialis, fever and deep venous thrombosis. SCT led to a complete hematological response, and marked improvement of the autonomic complaints, regaining of bowel control and normalization of blood pressure regulation, which allowed the patient to regain independence in the activities of daily life. Nerve conduction studies did not change after transplantation, and motor scores decreased slightly (from 140 to 137 out of 140), while the sensory sum score improved from 44 to 46 out of 56.

Patient 5 had a rapidly progressive sensory and motor demyelinating neuropathy and an IgG monoclonal gammopathy. She presented to the outpatient clinic with distal weakness in both legs, areflexia, hypesthesia of the fingers and lower legs, ataxia and very severe neuropathic pain, which did not respond to adequate dosage of either antidepressants or anticonvulsants registered for neuropathic pain treatment. The nerve conduction studies fulfilled the criteria for demyelination, and the diagnosis of CIDP with IgG monoclonal gammopathy was made. Despite repeated IVIg infusions, there was a severe progression of the neuropathy (Rankin scale 3). Treatment with high-dose dexamethasone and melfalan led to a further exacerbation of the polyneuropathy. After autologous stem cell transplantation, preceded by adriamycin/cyclophosphamide and melfalan cycles, no further progression occurred and the neuropathic pain disappeared, although motor scores did not improve. SCT was complicated by neutropenic fever.

Patient 6 had MM with IgD paraproteinaemia and a polyneuropathy with painful paresthesia of the feet, mild weakness of the M. extensor hallucis longus and intact reflexes. Nerve conduction studies were consistent with an axonal polyneuropathy. One year after the first sensory complaints she underwent autologous SCT, primarily aimed at the MM. SCT was complicated by herpes simplex infection in the mouth, urinary tract infection and fluid overload. SCT led to diminished neuropathic pain, but motor, sensory sum scores and nerve conduction studies did not improve after transplantation.

## Discussion

Here we report on the effect of stem cell transplantation on neuropathy in our cohort of 225 patients with monoclonal gammopathy and neuropathy. We retrospectively selected and analyzed six patients from this cohort who underwent SCT, primarily aimed at the malignancy underlying the monoclonal gammopathy rather than at the neuropathy. In only one patient, who received SCT for MM, did the polyneuropathy improve objectively. In one other patient, treated with SCT for primary amyloidosis, autonomic symptoms improved subjectively, allowing him to regain independence in the activities in daily life; in three other patients the neuropathy was at best stable after SCT, with further decline in motor function, but improvement in reported pain and in one patient slight improvement in sensory sum scores. However, one patient died after as a complication of the SCT. These results suggest that SCT halts progression in a minority of cases of polyneuropathy associated with B-cell dyscrasias, but that concerns about safety need to be addressed.

Efficacy of SCT for refractory polyneuropathy associated with B cell dyscrasia has not been studied systematically. Case reports have documented improvement after SCT in some patients with CIDP (7 patients), POEMS (42 patients) [5–8] and most recently in four patients with MGUS associated neuropathy [10], but publication bias may have precluded reports of cases with an unfavorable outcome. SCT results ranged from complete remission of the neuropathy in some POEMS and CIDP patients to full relapse after initial improvement, and unresponsiveness to SCT. In comparison to the patients described in previous studies, all our patients but two had an axonal polyneuropathy. This may explain the relative lack of beneficial effect of stem cell transplantation in our group compared to the recent publication of SCT for MGUS associated neuropathy, as all patients in that study had a demyelinating neuropathy.

The potentially beneficial effect of stem cell transplantation needs to be carefully weighed against the considerable risks of treatment-related mortality and morbidity. The European Group for Blood and Marrow Transplantation (EBMT) registry has reported transplantation-related mortality in 5% of 900 patients with autoimmune disease between 1995 and 2007, with mortality clearly improving in the years covered by the registry [14]. The treatment center and the underlying disease may also represent determinants of mortality due to SCT complications. In a recent study, 2 out of 13 patients (15%) with AL amyloidosis and autonomic neuropathy died within a 100 days after transplantation and were considered transplantation-related deaths [9]. Results from more studies are needed to document whether SCT for B cell dyscrasias associated with polyneuropathy carries a greater than average risk for complications and death. The case history of patient 3 emphasizes that the natural disease course of some B cell dyscrasia-associated neuropathies is relentless, which justifies treatment strategies with a higher risk for complications.

Due to the small number of patients with B cell dyscrasia associated neuropathy who underwent SCT, we could not establish an association between efficacy of SCT and the underlying hematological disease. The case history of patient 5 confirms findings from previous studies that autologous SCT can be considered as treatment of last resort in patients with CIDP and an associated monoclonal gammopathy, even if the B cell dyscrasia by itself does not warrant such treatment. Unfortunately, clinical trials establishing whether and when autologous stem cell transplantation should be considered are probably not feasible due to the small numbers of eligible patients. Standardized criteria of efficacy and detailed descriptions of patients treated with SCT may help to clarify whether specific nerve conduction features (i.e., demyelination, axonal loss) or underlying hematological disorder may predict outcome.

In conclusion, SCT might be considered as a treatment of last resort in very severe, progressive and therapy-resistant polyneuropathies associated with B cell dyscrasias. Nevertheless, the treatment-related mortality and morbidity and the relatively high chance of a limited response at best necessitate a careful and critical weighing of risks and benefits in each individual patient, and treatment should be limited to centers that have extensive experience with SCT and very low mortality rates.
